# The Effect of Androgen Deprivation Therapy on the Cardiovascular System in Advanced Prostate Cancer

**DOI:** 10.3390/medicina60111727

**Published:** 2024-10-22

**Authors:** Allison B. Reiss, Samantha Vasalani, Jacqueline Albert, Wendy Drewes, Kathleen Li, Ankita Srivastava, Joshua De Leon, Aaron E. Katz

**Affiliations:** 1 Department of Medicine and Foundations of Medicine, NYU Grossman Long Island School of Medicine, Mineola, NY 11501, USA; sev1222@live.com (S.V.); jacqueline.albert@duke.edu (J.A.); wendy.drewes@nyulangone.org (W.D.); ankita.srivastava@nyulangone.org (A.S.); joshua.deleon@nyulangone.org (J.D.L.); 2 Department of Urology, NYU Grossman Long Island School of Medicine, Mineola, NY 11501, USA; katie_li@urmc.rochester.edu (K.L.); aaron.katz@nyulangone.org (A.E.K.)

**Keywords:** androgen deprivation therapy, cardiovascular, advanced prostate cancer, hormonal therapies, management strategies, testosterone

## Abstract

Androgen deprivation therapy (ADT) is a mainstay treatment for metastatic prostate cancer, improving progression-free survival. ADT suppresses the production of testosterone and reduces circulating levels of the hormone. Luteinizing hormone-releasing hormone (LH-RH) agonists are the most commonly used ADT modality. They can be given alone or in combination with androgen synthesis inhibitors or androgen receptor antagonists. An estimated 40% of prostate cancer patients will receive ADT as part of their therapy during their lifetime. However, ADT has numerous adverse effects, including an increased cardiovascular risk that impacts quality of life. Relugolix is an alternative form of ADT. It is the only oral gonadotropin-releasing hormone antagonist, circumventing injection site reactions, making it easier for patients to take, and thus increasing compliance. Testosterone suppression with relugolix is excellent and testosterone recovery after discontinuation is rapid. This paper reviews the ADT and anti-androgen treatment options for men with prostate cancer and the cardiovascular effects of these therapies. There is accumulating evidence that cardiovascular risk with relugolix is lower than with other ADT medications and also lower than with androgen synthesis inhibitors and androgen receptor antagonists. This paper provides insight into the use of different ADT regimens based on the cardiovascular status and circumstances. It explores strategies to mitigate negative cardiovascular consequences and highlights the need for further study.

## 1. Introduction

Prostate cancer is the most common non-cutaneous cancer in men, with over 1.4 million new cases reported globally in 2020 [1,2]. The estimated lifetime risk of males developing prostate cancer is 12.5% in the United States and 4.65% globally [1,3]. However, many of these millions of patients can live for years with prostate cancer. Recent US Cancer Statistics data shows an overall 10-year survival rate of 97%; even in cases of prostate cancer with distant metastasis, patients had a 36% 5-year and 19% 10-year survival rate [4,5,6]. A significant proportion of these patients manage their cancer with androgen deprivation therapy (ADT) [7]. A 2016 survey found that 38% of patients globally with non-metastatic prostate cancer received ADT, and a 2002 study estimated that ADT was prescribed in 45% of all prostate cancer cases [8,9,10]. Due to the magnitude of people impacted and their length of survival, there has been growing interest in the side effects of ADT, especially the increased risk of cardiovascular disease (CVD). This review, therefore, examines overall associations between ADT use and CVD risk, explores underlying molecular mechanisms, and discusses approaches to reduce CVD risk in prostate cancer patients treated with ADT. Additionally, this review will focus on relugolix, a new form of ADT that is a gonadotropin-releasing hormone (GnRH) antagonist, in comparison to the commonly used GnRH agonist ADT medications.

## 2. Treatment Options for Prostate Cancer

Prostate cancer treatment options depend on whether the disease is localized or metastatic. For localized prostate cancer, the options include radical prostatectomy, prostate brachytherapy, external beam radiation, or active surveillance [11]. Relugolix may be part of the regimen in this population for those men at higher risk and requiring radiation [12,13,14]. In metastatic prostate cancer, the treatment options include chemical castration with antiandrogens, GnRH agonists, or GnRH antagonists, or in rare cases, surgical orchiectomy [15,16].

## 3. Cardiovascular Risk with ADT Therapy

CVD is the leading non-cancer cause of death in men with prostate cancer, with 32% of deaths attributed to CVD [17,18]. ADT, a valuable treatment option for men with local and metastatic prostate cancer, has been surrounded with contradicting evidence regarding the cardiovascular risk and endpoints [19,20,21]. The American Heart Association, The American Cancer Society, and the American Urological Society in 2010 released a joint statement that the evidence for increased cardiovascular risk in men with prostate cancer outweighs evidence against it, raising awareness of the association of cardiovascular risk with ADT treatment for both the clinician and patient [22]. A growing body of evidence from observational and randomized clinical trials demonstrates that despite excellent ADT treatment outcomes, the use of ADT has been associated with increased cardiovascular risk for these patients [23,24,25,26,27]. It has been estimated that two-thirds of men with prostate cancer are at increased risk for CVD [28].

Higher rates of cardiovascular events and specifically acute myocardial infarction, non-fatal stroke, myocardial infarction, and heart failure, as well as thrombotic and arrhythmic events, and acquired QT interval lengthening have been reported [29]. Moreover, patients with a pre-existing history of heart failure, myocardial infarction, or arrhythmia may be at an elevated risk of cardiovascular hospitalization and cardiovascular mortality when treated with ADT [30,31]. Some evidence suggests that the risk of cardiovascular events occurring within six months after the initiation of ADT is especially high, but other studies did not find this relationship [32,33]. CVD risks associated with ADT may require underlying conditions such as diabetes or hypertension to manifest [34,35]. Additionally, there remains disagreement on the impact of these risks during the first six months of treatment [36,37].

Men with prostate cancer and men with CVD share a number of similar risk factors, notably smoking, obesity, older age, and dyslipidemia [38,39,40]. ADT use in prostate cancer patients can cause metabolic derangements such as insulin resistance, dyslipidemia, diabetes, and obesity, thus contributing to this similar risk profile [41,42]. Multiple studies suggest that metabolic pathophysiologies, both those pre-existing in men within the prostate cancer population and those that emerge with the use of ADT, are causative factors of increased CVD risk and adverse CVD outcomes [43].

Metabolic syndrome with characteristics that include insulin resistance, glucose intolerance, hyperinsulinemia, increased very-low-density lipoprotein, hypertriglyceridemia, decreased high-density lipoprotein and hypertension have been noted in this population [44,45,46]. Lowering testosterone levels causes changes in the body composition such as increases in subcutaneous and visceral fat and decreases in lean muscle mass that can contribute to insulin resistance and CVD risk [47,48,49]. It should be noted that some studies do not find an association between ADT and insulin resistance [50]. A testosterone deficiency has also been noted to increase the risk of arrhythmias [51,52].

**Figure 1:**
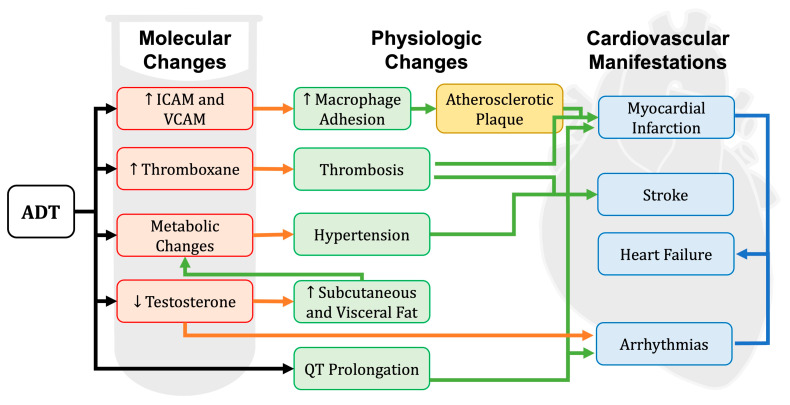
ADT initiates a number of interrelated molecular and physiologic changes associated with increased cardiovascular risk. Increased expression of adhesion molecules and thromboxane promote clotting while metabolic disruptions and loss of testosterone interact to raise blood pressure and increase adiposity. QT prolongation elevates the risk of arrhythmia. Ultimately, these changes may contribute to multiple forms of cardiovascular disease, including myocardial infarction, stroke, heart failure, and arrhythmias. ↓ = decrease Abbreviations: ADT—androgen deprivation therapy; ICAM—intercellular adhesion molecule; VCAM—vascular cell adhesion molecule.

Changes in the immune and endothelial function occur with prostate cancer and with ADT which can increase endothelial adhesiveness with the production of ICAM and VCAM [53,54,55]. Circulating thromboxane levels also rise with ADT [56]. Figure 1 provides an overview of the cardiovascular consequences of ADT.

## 4. Cardiovascular Risk Differences with Gonadotropin-Releasing Hormone Agonists Versus Antagonists

The relative cardiovascular risk and adverse cardiovascular events appear to be different when comparing types of ADT treatments, specifically GnRH agonists and antagonists.

The mechanisms of action that are contributory to the increased risk associated with GnRH agonists is not clear. Several hypotheses regarding the unique mechanisms of action have been studied. These include testosterone fluctuations with ADT therapy, the level of the suppression of the follicle-stimulating hormone (FSH), and immune system alterations that promote arterial plaque instability [54,55,57]. The reduction in testosterone levels with use of both GnRH agonist and antagonist therapies is associated with a wide range of metabolic effects. The mechanism of the suppression of the FSH with ADT therapy when using an agonist versus an antagonist differs between the two. GnRH agonists drive an initial overstimulation of GnRH receptors with a transient surge in the FSH, while antagonists do not drive an FSH surge [58]. The initial activation of the GnRH receptors by agonist treatment is followed by desensitization and a reduced LH and FSH release. In contrast, GnRH antagonists bind to and block the receptors, directly causing a sustained decrease in FSH and LH secretion. Murine studies have shown the relationship between low testosterone, the FSH, and atherosclerosis [59,60]. FSH secretion is associated with endothelial dysfunction and pro-atherogenic pathophysiologies [54]. In Apolipoprotein E-deficient mice vulnerable to atherosclerosis, preventing the GnRH agonist-induced surge in the FSH using an anti-FSH antibody reduced the atherosclerotic lesion area [53]. The FSH has been associated with cardiovascular risk determinants of metabolic derangements and atherosclerotic plaque formation such as the promotion of insulin resistance and weight gain [61,62,63]. This evidence potentially provides rationale for further mechanistic study on FSH secretion [64]. However, a population level study from Durham, North Carolina, USA, found that pre-treatment FSH levels were not associated with cardiovascular outcomes after ADT treatment [65].

Another cardiovascular danger from ADT is the prolongation of the QT interval on the electrocardiogram. This effect is seen with both GnRH agonists and antagonists and is thought to be a result of medical castration [66]. Hasegawa et al. found that the QT interval length increased in about 80% of prostate cancer patients given ADT (119 of 149 patients) [67]. The life-threatening elevated risk of the arrhythmia torsades de pointes, which can degenerate into ventricular fibrillation, is the major concern with QT prolongation, and in this study, torsades de pointes occurred in 1.3% of patients.

Numerous studies, including retrospective cohort studies, population-based cohort studies, meta-analyses, and randomized clinical trials, have produced consistent evidence that the CVD risk is higher with GnRH agonist compared to antagonist treatments [34,68,69,70]. A retrospective cohort study of 9785 prostate cancer patients who received ADT showed that the incidence of cardiovascular events was significantly higher in the GnRH agonist treatment group when compared with the GnRH antagonist treatment group [71].

A retrospective study used pooled data on 2328 men with prostate cancer from six phase III randomized trials to compare the CVD effects of the GnRH agonist versus antagonist treatment [72]. A total of 1491 patients received a GnRH antagonist while 837 received an agonist, and the CVD history was about 30% in both treatment groups. In the subgroup of men who had a pre-existing history of CVD, the risk of major cardiac events in the first year post-treatment was almost halved in men treated with the GnRH antagonist in comparison to the GnRH agonist. The pre-existing cardiovascular conditions considered in this study included myocardial ischemia, coronary artery disease, previous myocardial infarction, a cerebrovascular accident, a history of coronary artery bypass, and angina pectoris.

Margel et al. performed a randomized, open-label phase II study of CVD risk in adult males with prostate cancer who had already experienced a prior CVD event [34]. The study patients were randomized to receive either a GnRH antagonist or agonist. At one year, the endothelial function was compared between the treatment groups as the primary outcome measure and cardiovascular events as a predefined secondary outcome. While the endothelial function did not differ between the groups, 20% of the patients receiving the GnRH agonist experienced a major cardiovascular and cerebrovascular event while only 3% of those given a GnRH antagonist experienced an event (*p* = 0.013). The risk was reduced by 18% in the GnRH antagonist arm of the study.

Cone et al. analyzed the association between the GnRH agonists versus antagonists and the overall cardiovascular events and specific cardiac events, such as myocardial infarction and heart failure, using data available in the World Health Organization database of individual case reports of adverse drug reactions [73]. This source encompasses data from over 130 countries. They found a significant relationship between GnRH agonist drugs and overall cardiac events as well as myocardial infarction and heart failure, while GnRH antagonists only had a signal for heart failure. The onset of cardiac events related to agonists averaged in excess of 1 year.

## 5. Cardiovascular Impacts of Relugolix

The implementation of GnRH antagonists such as relugolix in treating prostate cancer has shown promising preliminary results in mitigating cardiovascular risk in comparison to GnRH and GnRH agonists [74,75]. Still, there exist several cardiovascular risks for patients taking orally prescribed relugolix.

Relugolix, like other ADT medications, can incite electrical changes in the heart, including QT prolongation [51,76]. The prolongation of the QT interval on the electrocardiogram is associated with a higher risk of developing torsades de pointes, which can progress to pulseless ventricular tachycardia, cardiac arrest, and sudden cardiac death [77,78].

Relugolix was specifically compared to leuprolide, an injectable GnRH agonist commonly used to treat prostate cancer, in both their castration efficacy and cardiovascular risk in the HERO trial [79]. Leuprolide can be administered either subcutaneously or intramuscularly [80]. The HERO trial is a Phase III, randomized, open-label trial that excluded patients who had experienced a major cardiovascular event in the 6 months prior to enrollment. This trial found a higher sustained castration rate for relugolix compared to leuprolide (96.7% versus 88.8%). The study found that 96.7% of patients that received an oral dose of 120 mg of relugolix once daily sustained castration throughout 48 weeks, while 88.8% of patients receiving leuprolide injections every 3 months maintained castration levels. Testosterone suppression to castration levels (<50 ng/dL) was reported from day 29 through to week 48 in patients receiving relugolix, which was non-inferior to the leuprolide results by an aforementioned seven points [81]. This superiority of relugolix was accompanied by a 2.9% incidence of major adverse cardiovascular events, compared to 6.2% in the leuprolide treatment group. When compared to leuprolide, relugolix allowed for the more rapid and complete restoration of testosterone levels upon discontinuation [79,82]. Quality of life, measured using validated questionnaires, was comparable between the two drugs [83]. Based on the HERO trial results, relugolix became the first FDA-approved oral GnRH antagonist for advanced prostate cancer treatment [84,85,86].

In comparison to injectable GnRH antagonists, relugolix has been shown to have increased efficacy, with no injection site reaction to consider due to its availability as an oral medication [85]. A network meta-analysis encompassing four phase III randomized trials and 2059 patients found that relugolix outperformed degarelix, an injectable GnRH agonist, in all efficacy ranking analyses, and achieved similar safety scores for both overall adverse events and cardiovascular event rates [87,88,89,90,91]. Both relugolix and degarelix received superior safety scores for cardiovascular event rates in comparison to GnRH agonists. In contrast to relugolix and leuprolide, degarelix is not associated with QT prolongation [92,93]. It has been noted that degarelix is associated with reports of more frequent injection site reactions and is generally given only by a healthcare professional in a clinical setting [94,95]. The half-life of relugolix is shorter than that of degarelix, leading to more rapid testosterone recovery [96,97,98]. The authors note that it is unclear whether rapid testosterone recovery would be beneficial to patient outcomes, due to a rapid return to physiological normality, or harmful to patient outcomes, as testosterone aggravates tumorigenesis. The ability to administer the medication without a clinical visit is an additional advantage to an oral drug. Travelling to a clinic or office is a complication for many patients who do not have access to private transportation or for patients who work full-time jobs. Though medical provider concerns about patient adherence to daily medication cannot be dismissed, it has been suggested that there is a patient preference for the oral over the intravenous route [99].

The main similarities and differences in the properties of relugolix, degarelix, and leuprolide are summarized in Table 1.

## 6. Approaches to Minimize Cardiovascular Risk in Prostate Cancer Patients Treated with ADT

Given the prevalence of CVD in prostate cancer patients, some precautions can be taken to mitigate the cardiovascular risk during prostate cancer treatment. The recognition of the issue is key since prostate cancer patients tend to be under-evaluated for cardiovascular health and have a low compliance rate with treatment plans for mitigating CVD risk [100,101,102]. A joint statement from the American Heart Association, American Cancer Society, and American Urologic Association recommends a cardiologic evaluation of patients beginning ADT, including an assessment for cardiovascular risk factors such as blood pressure, glucose levels, and lipid profile [22]. This statement does not recommend specific testing or coronary intervention prior to ADT initiation, since there is currently no data to support that stress testing or revascularization can prevent future CVD risk.

Other statements, such those released by the European Association of Urology (EAU), and from the Hong Kong Urological Association (HKUA), and the Hong Kong Society of Uro-Oncology (HKSUO), similarly recommend baseline CVD screenings [103,104]. However, a large cross-sectional analysis of men treated for prostate cancer in the US Veterans Health Administration found that there was a high rate of patients not evaluated nor treated for cardiovascular risk factors; similarly, in a small survey of physicians treating prostate cancer patients with ADT, only 36% reported screening for hypertension and 64% reported initiating treatment for CVD [102,105]. This highlights the need for further physician education and awareness about the proper management of CVD and CVD risk in patients with prostate cancer.

There is a consensus among healthcare experts that patients with pre-existing cardiovascular conditions or those with several risk factors for adverse cardiac events should be carefully monitored as they undergo ADT, but it is estimated that only about 60% of those undergoing ADT have their CVD risk factors fully documented [39]. The INTERHEART study established nine modifiable risk factors for adverse cardiovascular events in 27,098 women and men that increase their susceptibility to myocardial infarction. These are hypertension, smoking cigarettes, abdominal obesity, high stress levels, diabetes, alcohol use, a sedentary lifestyle, abnormal lipid profile, and poor diet with a high saturated fat and salt content [106,107]. Patients with these pre-existing risk factors should be evaluated and categorized based on the degree of CVD risk they present to ensure that proper precautions are taken for susceptible prostate cancer patients [108,109]. Furthermore, several techniques have been proposed to establish baseline CVD and CVD risk. The HKUA and HKSUO joint statement includes recommendations for cardiac imaging studies such as electrocardiograms, transthoracic echocardiograms, or multigated acquisition scans to establish the baseline risk [103]. Coronary artery calcium scores may also be applied as part of a thorough evaluation of CVD risk in prostate cancer patients [110]. Some limited evidence suggests that serial myocardial perfusion PET scans may also be useful to detect and monitor coronary artery disease and coronary microvascular dysfunction in prostate cancer patients taking ADT. Welch et al. conducted a prospective cohort study in prostate cancer patients with a high CVD risk based on the Framingham Score using serial myocardial perfusion PET scans with the first imaging completed within 3 weeks of the start of ADT with a repeat after 6 months, and found this to be a very sensitive way to detect coronary artery disease and coronary microvascular dysfunction [111]. Overall, there is agreement that cardiovascular and renal parameters such as blood pressure, body mass index, hemoglobin A1C, uric acid, serum electrolytes, creatinine, and the lipid profile should be monitored in prostate cancer patients on ADT [103,112]. These lab values and risk factors together may be used to calculate a Framingham risk score, Atherosclerotic Cardiovascular Disease (ASCVD) risk score, or other comparable risk score. Patients with abnormal values or scans should be referred to a cardiologist or cardio-oncologist, endocrinologist, and/or general practitioner for follow-up.

Patient race and demographics should be considered during screening and monitoring procedures, as well as during treatment. For instance, patients of African descent experience both prostate cancer and CVD at younger ages, at higher rates, and with poorer outcomes than patients in other demographics [113]. Recent analyses show that African–American patients may also have the same or better survival outcomes when taking ADT in comparison to white patients, but are less likely to receive ADT or have a longer delay before starting ADT [114,115,116,117]. Disparities in the incidence and burden may be due to a combination of genetic, behavioral, environmental, and socioeconomic factors, with the last factor being the most significant. In studies that control for the socioeconomic status and in the setting of equal-access care systems, African–American and White patients have similar mortality rates [118,119]. Still, prostate cancer may be associated with different molecular profiles in African–American vs. White patients, which has the potential to influence their response to cancer therapies [120]. In addition to race and ethnicity, the geographic location may also be associated with differences in ADT usage. For example, men residing in the south had a longer time before ADT initiation in comparison to men in the northeast, and men with low-risk prostate cancer residing in the mid-west, south, and northeast were more likely than men in the west to receive ADT despite the recommendations against ADT prescription for low-risk prostate cancer [121]. More research is needed to better understand and address these racial and geographic disparities, but providers must consider them in counselling their patients.

To summarize these monitoring recommendations as well as recommended interventions, several authors have proposed the implementation of an ABCDE paradigm to reduce the risk of adverse cardiovascular effects in patients undergoing ADT [122]. The ABCDE paradigm seeks to decrease CVD risk in ADT patients by incorporating patient awareness, 81 mg aspirin daily, blood pressure parameters of <140/90 mm Hg, cholesterol control via statin therapy for pre-existing CVD, cigarette cessation, diabetes and blood glucose monitoring, a diet rich in fruits and vegetables with high vitamin D and calcium, and light exercise [29]. In addition to aspirin, other physicians have also recommended starting a P2Y12 inhibitor or an anticoagulant like rivaroxaban, with optional angiotensin-converting enzyme (ACE) inhibitors, angiotensin II receptor blockers (ARBs), and beta blockers as needed. The HKUO and HKSUO guidelines also elaborated on the exercise category, recommending over 6 months of combined aerobic and resistance training, with Bhatia et al. recommending a slow increase in exercise to a goal of 150 min per week of moderate exercise or 75 min per week of vigorous exercise [29,103]. It is worth noting that these principles align closely with guidelines for cardiovascular risk mitigation for the general public [106]. The inclusion of an additional “D” factor, diversity, in the ABCDE paradigm has been proposed to account for the different cardiovascular risk levels that are present in patients of different racial and ethnic backgrounds, as described above [23,123].

Care plans for patients at a high CVD risk are similar to these general guidelines, and include the close monitoring of cardiovascular parameters and the adoption of healthier lifestyle habits such as a heart-healthy diet and increased exercise. To ensure the increased CVD risk that occurs in patients undergoing ADT is properly monitored, an interdisciplinary care team is ideal and would include oncologists, cardiologists, urologists, primary care physicians, and potentially endocrinologists for diabetic patients [124,125]. At the University of Texas MD Anderson Cancer Center, a Healthy Heart program was established to manage the cardiometabolic risk in prostate cancer patients through medical management and individually tailored exercise programs [126]. The success of this initiative was limited by the failure of patients to follow-up with their physicians, demonstrating that the importance of CVD risk must be explained to patients to make such a program impactful.

Continuing the use of cardioprotective medicine during prostate cancer treatment is important. Shore et al. performed a subgroup analysis looking at men enrolled in the HERO trial who were taking medications for CVD in the categories of antihypertensives, antithrombotics, and lipid-modifying agents and found that there were numerically more fatal adverse events in men on antithrombotic or lipid-lowering drugs and more cardiovascular-related fatal events in men on leuprolide than on relugolix [127]. Overall, the men on relugolix and cardiovascular medications tolerated the combination well and did not show any loss of relugolix efficacy with multiple drugs.

Other proposed interventions to minimize the cardiotoxicity of prostate cancer therapies include pre-treatment with cardiac medications such as anti-arrhythmic drugs, beta-blocker, or ACE inhibitors. This could help protect prostate cancer patients from adverse cardiovascular effects [128]. Some authors have suggested a combination of aspirin and low-dose rivaroxaban for high-risk patients, but these drugs come with their own potential adverse consequences such as bleeding in the gastrointestinal tract and elsewhere [129,130].

Treating patients with these powerful drugs inevitably brings the possibility of significant side-effects. Statins, competitive inhibitors of 3-hydroxy-3-methyl-glutaryl-CoA reductase, are highly effective in reducing serum low-density lipoprotein (LDL) cholesterol, but muscle pain is seen in over 20% of statin users. Muscle weakness can occur and, although rare, rhabdomyolysis has been reported [131]. Coenzyme Q10 supplementation can alleviate some of the muscle symptoms [132]. Statins may also cause liver enzyme abnormalities and increase the risk of diabetes [133,134]. The benefit of statins in lowering major adverse cardiovascular events is proportional to the degree and duration of LDL elevation [135].

Beta blockers act by impeding cardiac beta-1 adrenergic receptor activation and thereby decreasing the blood pressure, heart muscle contractility, and oxygen demand [136]. Beta blockers are known to cause erectile dysfunction and depression, which are already issues for many men during prostate cancer treatment [137]. Fatigue and weight gain are also commonly seen with beta blockers. Due to all these negatives, the discontinuation rates for this drug class are high. They are most effective in persons with heart failure and a low ejection fraction [138].

ACE inhibitors are effective in treating hypertension and heart failure [139]. They cause vasodilation, reduce systemic vascular resistance, and attenuate endothelial dysfunction. The side-effects associated with ACE inhibitors include a dry cough, hyperkalemia, and, more rarely, liver toxicity and angioedema [140,141].

Further research is needed to determine the efficacy of adding cardiac medications to prostate cancer treatment plans, though the preliminary data have been somewhat promising.

Intermittent treatment with pauses to allow testosterone levels to recover may reduce CVD event rates [142]. A recently published commentary on cardiovascular risk in prostate cancer calls attention to the need for more effort on the part of healthcare professionals in CVD risk assessments and earlier, more aggressive, and unvarying follow-through on improving modifiable risk factors [143]. The equivalence of the data across different types of studies showing less of a CVD risk with GnRH antagonists relative to agonists may factor into the choice of treatment with consideration of the CVD risk profile [70,71,144,145].

## 7. Future Directions

Awareness in the healthcare community of the association between ADT and cardiovascular events is an imperative first step in reducing the consequences [146]. Assessments for risk prior to the initiation of ADT therapy, particularly in those patients with pre-existing CVD conditions, should be undertaken. Aggressive, standardized lifestyle modification efforts, inclusive of medical management, exercise programs, heart-healthy dietary counseling, smoking cessation, guided medical therapy, and careful monitoring by multidisciplinary teams, may help to mitigate the risk. Additionally, the study of possible pretreatment with cardiac medications to reduce the cardiotoxicity of ADT therapies may provide a pathway for CVD risk reduction in prostate cancer patients receiving ADT. Rigorous controlled trials would allow the collection of validated evidence regarding the selection and timing of medications with the best risk profile for both CVD and prostate cancer outcomes.

Further research regarding the mechanism of action of GnRH agonists and antagonists to determine definitively how these agents are contributory to CVD risk is needed. Specifically, relugolix research to answer remaining questions about its mechanism of actions and the consequences of these mechanisms, as well as for further confirmation of positive outcomes with its use, is needed. The endothelial dysfunction associated with ADT may be a viable focus for therapy [147].

A new addition to the armamentarium of GnRH antagonists is teverelix, a decapeptide now in clinical trials to assess the possibility that it will exhibit superior cardiovascular safety in prostate cancer patients [148,149]. Another possibility is to circumvent the need for GnRH agonists or antagonists by using estrogen to suppress testosterone production. In past studies with oral estrogen, the CVD risk was increased due to the hepatic metabolism leading to thrombotic events [150]. However, estrogen delivery via a transdermal patch bypasses the liver and is being studied as a prostate cancer treatment option that would avoid cardiovascular toxicities and might also preserve bone health and reduce hot flashes [151,152,153].

## 8. Conclusions

Multiple therapies for the treatment of local and metastatic prostate cancer are currently available. Treatment with GnRH agonists or antagonists improve progression-free survival. However, this positive response is accompanied by an increase in cardiovascular events, impelling efforts to raise awareness of CVD risk for patients undergoing ADT. There are known similarities in CVD risk profiles in prostate cancer and cardiovascular patients. Metabolic abnormalities noted to pre-exist or to emerge with ADT are possible causative factors for CVD events and risk. Changes in the immune and endothelial function that occur with prostate cancer and ADT may also be responsible. Further, CVD risk differences have become evident with types of ADT treatment. The oral GnRH antagonist relugolix is showing some promise in mitigating CVD risk compared to GnRH agonists. Further, it offers convenience and alleviates concern for injection site reactions. As more data become available and with new therapies emerging, high-quality evidence from clinical studies will aid in the optimal selection of the drug regimen for prolonged survival with the maintenance of cardiovascular health.

## Figures and Tables

**Table 1:** Comparison of basic clinical and pharmacologic features of relugolix, degarelix, and leuprolide.

Characteristics	Relugolix	Degarelix	Leuprolide	References
Type	GnRH antagonist	GnRH antagonist	GnRH agonist	
Route of Administration	Oral	Subcutaneous injection	Intramuscular or subcutaneous injection	[12,15,80,85,90]
Self-Administration	Yes	Must be given by a healthcare professional	Possible, but may require clinic visit	[12,15,79,84,90,94,95]
Frequency of administration	Daily	Monthly	1, 3, 4, or 6 months, depends on formulation	[75,79,80,88]
FDA approval	Approved in 2020 to treat advanced prostate cancer	Approved in 2008 to treat advanced prostate cancer	Approved in 1985 to treat advanced prostate cancer	[80,82,83,84]
Drug half-life	36–65 h	53 days	3 h	[80,85,88]
Efficacy	96.7% sustained castration throughout 48 weeks	97% sustained castration throughout 1 year	88.8% maintained castration levels throughout 48 weeks	[75,86]
Time to castration achievement	Median of 4–15 days	Median of 3 days	Median of 21 days	[81,96,97]
Median time to restoration of testosterone level after last dose	13 weeks (86 days) to >280 ng/dL	52 weeks to pretreatment level	16 weeks (112 days) to to >280 ng/dL	[82,98]
QT prolongation	Can cause QT prolongation	No QT prolongation	Can cause QT prolongation	[51,76,92,93]
Cardiovascular Event Rate	2.9%	5.5%	6.2%	[79,91]

FDA: Food and Drug Administration; GnRH: gonadotropin-releasing hormone.

## Data Availability

No new data were created or analyzed in this study. Data sharing is not applicable to this article.
